# A Hybrid Rao-NM Algorithm for Image Template Matching

**DOI:** 10.3390/e23060678

**Published:** 2021-05-27

**Authors:** Xinran Liu, Zhongju Wang, Long Wang, Chao Huang, Xiong Luo

**Affiliations:** 1 School of Computer and Communication Engineering, University of Science and Technology Beijing, Beijing 100083, China; G20208816@xs.ustb.edu.cn (X.L.); g20198877@xs.ustb.edu.cn (Z.W.); chao.huang@my.cityu.edu.hk (C.H.); xluo@ustb.edu.cn (X.L.); 2 Shunde Graduate School, University of Science and Technology Beijing, Foshan 528300, China; 3 Beijing Key Laboratory of Knowledge Engineering for Materials Science, Beijing 100083, China

**Keywords:** image matching, Rao algorithm, computational intelligence, optimization

## Abstract

This paper proposes a hybrid Rao-Nelder–Mead (Rao-NM) algorithm for image template matching is proposed. The developed algorithm incorporates the Rao-1 algorithm and NM algorithm serially. Thus, the powerful global search capability of the Rao-1 algorithm and local search capability of NM algorithm is fully exploited. It can quickly and accurately search for the high-quality optimal solution on the basis of ensuring global convergence. The computing time is highly reduced, while the matching accuracy is significantly improved. Four commonly applied optimization problems and three image datasets are employed to assess the performance of the proposed method. Meanwhile, three commonly used algorithms, including generic Rao-1 algorithm, particle swarm optimization (PSO), genetic algorithm (GA), are considered as benchmarking algorithms. The experiment results demonstrate that the proposed method is effective and efficient in solving image matching problems.

## 1. Introduction

Image matching is an important topic in image processing, and it has broad application prospects in the field of computer vision. Image matching typically includes Template Matching (TM), Feature Matching, and Dynamic Pattern Matching, among which TM is the most commonly used matching approach. TM is employed to measure whether an image patch matches a small area of the source image by sliding the template through the source image, and then use the coordinates of the upper-left corner of the corresponding window in the two images to determine the matching position [1].

TM is a fundamental problem of pattern recognition and has a wide range of applications in the field of image processing and computer vision, such as image recognition [2,3,4,5], remote sensing [6,7], social media analytics [8,9], medical image processing [10,11,12], biometric recognition [13,14,15], etc. In image analysis, matching technologies play an important role in image understanding and retrieval [16]. Two main operations, similarity measurement and best matching search [17,18] are often included in TM. Various similarity metrics are utilized to measure the similarity of two grayscale images, including Mean Absolute Differences (MAD), Sum of Absolute Differences (SAD), Sum of Squared Differences (SSD), and Mean Square Differences (MSD). Among these similarity measures, the normalized cross correlation (NCC) is commonly used for image matching, due to its robustness for the illumination variance and noise [19,20,21]. The NCC effectively reduces the influences of illumination on image comparison results, and it is more suitable for processing images with slightly deformed objects, blurred or unclear images, and textured images.

The full, exhaustive search algorithm [22] is the simplest TM approach. It can check each pixel candidate at once and has extremely high accuracy. However, this kind of exhaustive search has an extremely expensive computation cost because every pixel of the source image has to be compared with NCC values computed, which severely limits its use in image processing applications [23]. In this paper, to reduce the time of NCC computation and speed up image matching, TM algorithms based on computational intelligence algorithms were proposed in the literature.

Computational intelligence algorithms were extensively used for different optimization problems in previous studies. He et al. [24] developed a robust fuzzy programming approach to solve the multiple response optimization issues. Chen et al. [25] proposed an adaptive gradient method to ensure both the convergence and the communication efficiency of federated learning. Tang et al. [26] proposed an improvement in the stochastic optimization of the imaging inverse problems. Recently, the hybrid computational intelligence algorithms were developed and applied in various domains [27,28,29]. Computational intelligence-based algorithms were also employed in the area of image matching. Yan et al. [30] introduced the isolation niche technology into the traditional Cultural Algorithm (CA) and applied it to the image matching problem to improve stability and convergence precision. Liu et al. [31] proposed a Chaotic Quantum-behaved Particle Swarm Optimization Based on Lateral Inhibition (LI-CQPSO), which utilized the Chaos theory to ensure the PSO avoids premature convergence. Luo et al. [32] proposed a hybrid spotted hyena optimizer based on LI, which was applied for image pre-processing to make an intensity gradient in the image contrast-enhanced and enhanced the characters of the image. Huang et al. [33] discussed a hybrid bio-inspired evolutionary optimization approach incorporating the lateral inhibition mechanism and Imperialist Competitive Algorithm (ICA), addressing the limitation that the traditional ICA method is possibly trapped in the local minimum.

The above-mentioned methods often include algorithm-specific parameters, such as the cognitive and social factors in PSO, and tuning these parameters introduces additional computational cost. Meanwhile, they typically employ the correlation value as the fitness function to find the best matching point in the image through multiple iterations, thereby reducing the number of explorations and shortening the search time. However, these methods cannot search the entire solution space efficiently and are easy to converge prematurely. Therefore, they often fall into the optimal local state and miss the accurate position, resulting in low search precision and accuracy.

To address these limitations, a hybrid Rao-NM algorithm that combines the Rao-1 algorithm and the Nelder–Mead algorithm is proposed for the TM problem in this paper. The Rao-1 algorithm does not contain any algorithm-specific parameters, and only simple mathematical operations, addition, and multiplication, are included. The proposed method contains two search processes, global search, and local search. The Rao-1 algorithm is employed for the global search. The Rao-1 algorithm is a metaphor-less swarm intelligence method introduced by Rao [34] in 2019. The main idea of the Rao-1 algorithm is to iteratively update candidate solutions with the high probability of approaching the global best solution and leaving the worst solution. The optimal solution is obtained through the random interaction between the best and worst solutions. Meanwhile, the Rao-1 algorithm does not require any algorithm-specific parameters, and the computational cost of tuning parameters can be avoided. Recent research has proved its capability in solving different unconstrained and constrained optimization problems. During the local search process, the NM algorithm is utilized to further improve the search results of the Rao-1 algorithm. The NM algorithm is a popular nonlinear optimization search method without using derivative information introduced by Nelder and Mead [35,36]. The NM algorithm only considers function values to minimize the scalar-valued nonlinear function, without any derivative information [22]. It rescales the simplex of (n+1) vertices according to the local behaviors of the function through four basic processes: Reflection, expansion, contraction, and shrinkage. After these steps, the simplex can be self-improved and gradually approach to the optimal solution.

The rest of this paper is organized as follows. In Section 2, an optimization problem for TM is formulated. Section 3 presents the proposed hybrid Rao-NM algorithm. In Section 4, experiments and analyses are showed. Finally, the conclusion of this paper is provided in Section 5.

## 2. Problem Formulation

Image matching technologies are important in the field of airplane or missile map matching and positioning, medical image processing, and other related fields. The image matching process uses two sensors to get two images of different sizes from the same area. The image obtained in advance is called the source image, and the image obtained in real time or online during the matching process is called the template image. In this study, we use the NCC model [16] as the fitness function to compute the degree of matching between the template image and the source image and then determine the search position. Under the guidance of the fitness value, NCC coefficient, the hybrid algorithm can search the source image quickly until the area with the best similarity is found.

**Figure 1:**
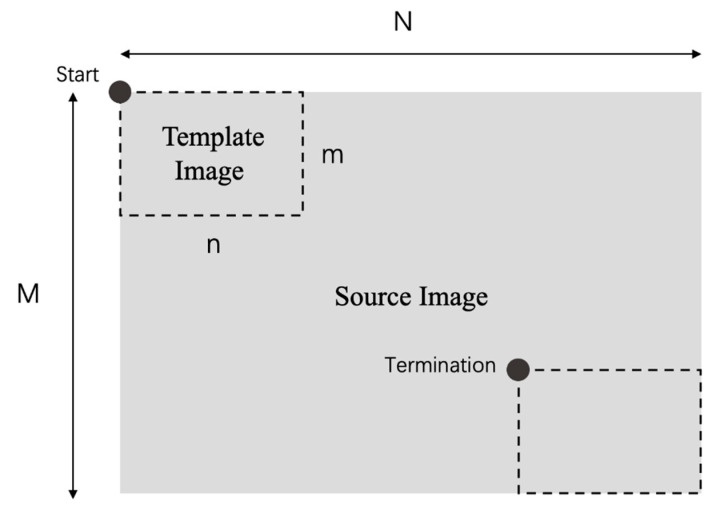
Template matching geometry.

Image TM aims to locate a small area of the source image by searching for a target similar to the template image by sliding the template through the source image, shown in Figure 1. To facilitate computation, both the template image and the source image are transformed to grayscale images. Let the matrix $X_{m\times n}$ and $Y_{M\times N}$ represent the grayscale template and source images, respectively, where *m* and *n* denote the height and width, and X[i,j] and Y[i,j] represent the gray values of a certain pixel of images, respectively (X[i,j], Y[i,j] ∈[0,255]).

The main idea of the TM problem is defined that search a point (x, y) in $Y_{a\times b},$ so that the similarity between X (1:m, 1:n) and Y (x:(x+m−1), y:(y+n)−1)) is the maximum in the feasible search space. The NCC metric can use the grayscale matrices of two images to compute the degree of matching between them through a normalized correlation measurement formula. Therefore, the TM problem can be presented as an optimization problem, depicted in (1).

(1) $$\begin{array}{ll} max\;F\left(i,j\right)= & \overset{m}{\underset{x=1}{\sum }}\overset{n}{\underset{y=1}{\sum }}\left[temp\left(i+x-1,j+y-1\right)\right]\times test\left(i,j\right)\\ & \;\times {\left(\sqrt{\overset{m}{\underset{x=1}{\sum }}\overset{n}{\underset{y=1}{\sum }}\left[temp^{2}\left(i+x-1,j+y-1\right)\right]}\;\cdot \sqrt{\overset{m}{\underset{x=1}{\sum }}\overset{n}{\underset{y=1}{\sum }}\left[test^{2}\left(i,j\right)\right]}\right)}^{-1} \end{array}$$

 s.t. 1≤i≤A−a+1, i∈Z, 1≤j≤B−b+1, j∈Z

where (i, j) is the pixel position of the top-left corner of the grayscale template matching, when the original image matches the same area as the template image at (i*, j*), NCC (i*, j*) = 1.

## 3. The Proposed Hybrid Rao-NM Algorithm

In this paper, we combine the Rao algorithm and the Nelder–Mead simplex method to efficiently obtain the optimal solution. In the proposed algorithm, the Rao-1 algorithm is employed for global search, while the NM algorithm is utilized to conduct the local search. In this section, the Rao-1 and NM algorithms are introduced separately, and then the hybrid Rao-NM algorithm is described in detail.

### 3.1. Rao-1 Algorithm

The Rao-1 algorithm is a metaphor-less swarm intelligence-based optimization method without containing any algorithm-specific parameters [34]. Only two controlling parameters, population size and the number of iterations, need to be determined for the Rao-1 algorithm.

The solution updating procedure of the Rao-1 algorithm is illustrated as (2) and (3):

(2) $${I^{\prime }}_{j,k,i}=I_{j,k,i}+r_{1,j,i}\left(I_{j,best,i}-I_{j,worst,i}\right)$$

(3) $$I\prime_{m,n}=\{\begin{array}{c} I\prime_{m,n}\;\;if\;F\left(I\prime_{m,n}\right)\leq F\left(I_{m,n}\right)\\ I_{m,n}\;\;if\;F\left(I\prime_{m,n}\right)>F\left(I_{m,n}\right) \end{array}$$

where $I_{j,best,i}$ is the value of the variable *j* for the best candidate and $I_{j,worst,i}$ is the value of the variable j for the worst candidate during the ith iteration. $I\prime_{j,k,i}$ are the updated values of $I_{j,k,i}$ and $r{\;}_{1,j,i}$ and $r{\;}_{2,j,i}$ are two random numbers of the jth variable during the ith iteration, with their value range in [0, 1].

Based on the updating rule, the optimization process of the Rao-1 algorithm is summarized as follows:

1. Initialize the common controlling parameters, population size, number of design variables, and termination criteria.
2. Determine the best and worst solutions in the population.
3. Update the current solution based on the best, worst, and candidate solutions, random interaction according to (2)
4. Computer the objective function value for every updated solution. Next, the updated solution will be selected according to (3).
5. If the termination conditions are satisfied, the optimization process will stop. Otherwise, the process skips to Step 2.

### 3.2. NM Method

The NM search method is a local search method, and it parameterizes the function value through unconstrained optimization without using the gradient information. The objective function shrinks to optimal value by adapting to the local landscape with simplex. Since the TM problem can be regarded as a two-dimensional optimization problem, a simplex is a triangle composed of vertices. If a point is defined as the origin of a non-degenerate simplex, the other *n* points will define the vector direction across the *N*-dimensional vector space [37].

NM method uses four basic steps to readjust the scale of the simplex according to the local behavior of the function: Reflection, expansion, contraction, and shrinkage [38]. The simplex can approach the optimal value continuously through these procedures.

Before starting the algorithm, defining the complete NM method requires four scaling parameters: Coefficients of reflection (α), contraction (γ), expansion (β), and shrinkage (σ). According to the definition of the NM method, these parameters should satisfy (4):

(4) α>0, γ>1, γ>α, 0<β<1, and 0<σ<1

As Image TM is actually a two-dimensional optimization problem, parameters are restricted to the standard case according to (5).

(5) $$\alpha =1,\;\gamma =2,\;\beta =\frac{1}{2},\;and\;\sigma =\frac{1}{2}$$

The specific steps of the NM method are described as follow:

1. Initialization:

Randomly Generate initial n+1 vertices within their respective search range. Compute the objective function value and the simplex constraint of each vertex, and then order these vertices to satisfy $f\left(x_{1}\right)\;\leq \;f\left(x_{2}\right)\leq \cdot \cdot \cdot \leq f\left(x_{n+1}\right)$.

2. Reflection:

Calculate the reflection point x_r_ according to the (6):

(6) $$\;x_{r}=\bar{x}+\alpha \left(\bar{x}-x_{h}\right)$$

where $\bar{x}=\sum_{i=1}^{n}\frac{\;x_{i}}{n}$, β*_h_* and β*_l_* are the vertices with the highest and lowest function values, respectively, $f\left(x_{h}\right)$ and $f\left(x_{l}\right)$ represent the value of the observation function. Next, obtain the x_c_, which is the center of the simplex without x_h_ in minimization case. If $f\left(x_{r}\right)<f\left(x_{l}\right),$ go to step 3; If $f\left(x_{r}\right)>f\left(x_{h}\right)$, go to step 4; otherwise, if $f\left(x_{r}\right)$ lies between $f\left(x_{l}\right)$ and $f\left(x_{h}\right)$, x*_h_* is replaced by x*_r_* and go to step 6.

3. Expansion:

To expand the search space in the same direction, the expansion point is expanded the simplex and computed as (7):

(7) $$x_{e}=\gamma x_{r}+\left(1-\gamma \right)x_{c}$$

If $f\left(x_{e}\right)<f\left(x_{r}\right)$, x_h_ is replaced by x_e_;

If $f\left(x_{e}\right)\geq f\left(x_{r}\right)$, x_h_ is replaced by x_r_;

Go to step 6.

4. Contraction:

When $f\left(x_{r}\right)$ lies between $f\left(x_{l}\right)$ and $f\left(x_{h}\right)$, then x_h_ is replaced by $x_{r}$ and contraction is performed. When $f\left(x_{r}\right)$>$\;f\left(x_{h}\right)$, perform contraction directly without any replacements. The contraction vertex is computed as follow (8):

(8) $$x_{cont}=\beta x_{h}+\left(1-\beta \right)x_{cent}$$

If $f\left(x_{cont}\right)$ < $f\left(x_{h}\right)$, $x_{h}$ is replaced by $x_{cont}$ and go to step 6. Otherwise, do shrinking in step 5.

5. Shrinkage:

When the contraction is failed, shrinkage attempts to all vertexes of the entire simplex expect *x_l_* as (9):

(9) $$x_{i}=\sigma x_{i}+\left(1-\sigma \right)x_{l}$$

Then go to step 6.

6. If the termination condition is met, the computation is stopped and terminates the iteration. Otherwise, return Step 1 to start a new iteration.

### 3.3. The Hybrid Rao-NM Algorithm

The Rao-NM algorithm combines the adaptive Rao-1 algorithm and the NM method to balance the efficiency and accuracy of the optimization process with a higher probability of obtaining the optimal solution within limited iterations.

In the optimization process, the Rao-1 algorithm [34] is initially applied to finding a relatively optimal solution, and the search space is reduced for the continued search. Next, according to the solution obtained from the Rao-1 algorithm, the NM method [35] is utilized to search the best local solution near the initial solution. Compared with the generic Rao-1 algorithm, the proposed hybrid algorithm can offer better solutions thanks to the NM method. Meanwhile, the Rao-NM algorithm can converge quickly, inheriting the advantage of the Rao-1 algorithm. The main optimization process of the proposed Rao-NM algorithm is described in Algorithm 1.

As shown in Algorithm 1, considering the multiplication operation of the NCC computation as the basic operation, the time complexity of the proposed algorithm is *O*(*M·N·w·h*), where *w* and *h* are the weight and height of the template image, respectively. Thus, it is independent of the size of the source image.
**Algorithm 1.** **Rao-NM Algorithm.**
1: Input: Population Size: *N*, Number of Iterations: *M*, Tolerance: *e*, The *i*th individual solution at the *j*th iteration: *I_i_*_,*j*_
2: Output: Optimal Solution: I*_best_
3: **for each**
*j*: = 1 to N **do**
4:    Initialize *I_i_*_,*1*_;
5: **end**
6: Let *j* = 1;
7: **While** (*e* or *j* value is satisfied)
8: Update solutions *I_i_*_,*j*_ based on (2);
9: Obtain the best solution *I_best_*;
10: Let *e* = *j*(*I_bes_*_t_);
11: Let *m* = *m* + 1.
12: Update *I_best_* via NM algorithm to I*_best_;
13: **Return**
I^*^_best_;

## 4. Experiment and Analysis

### 4.1. Benchmarking Test Functions

**Figure 2:**
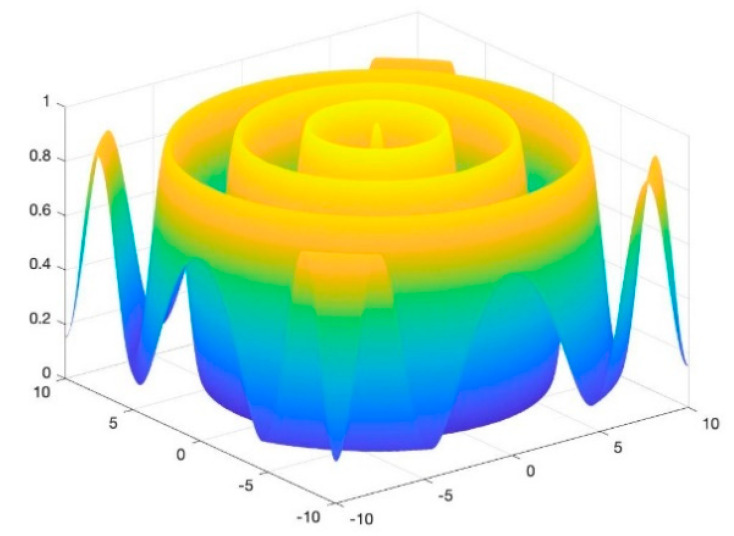
Image of Function 1.

**Figure 3:**
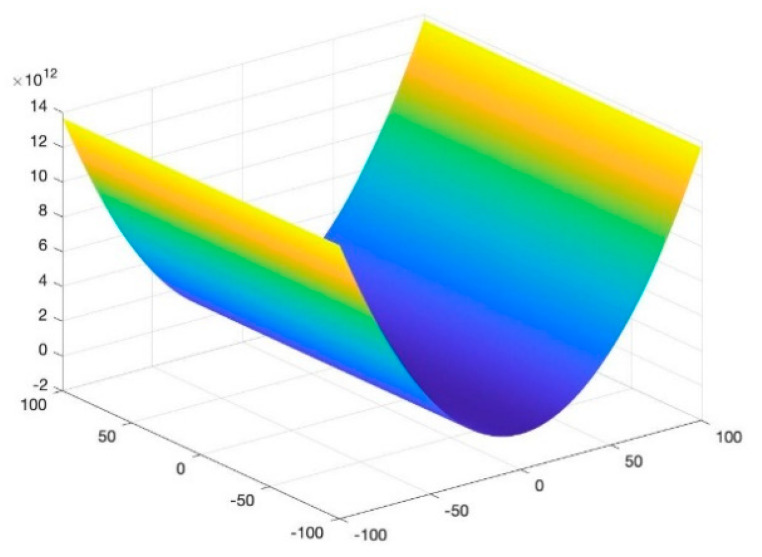
Image of Function 2.

**Figure 4:**
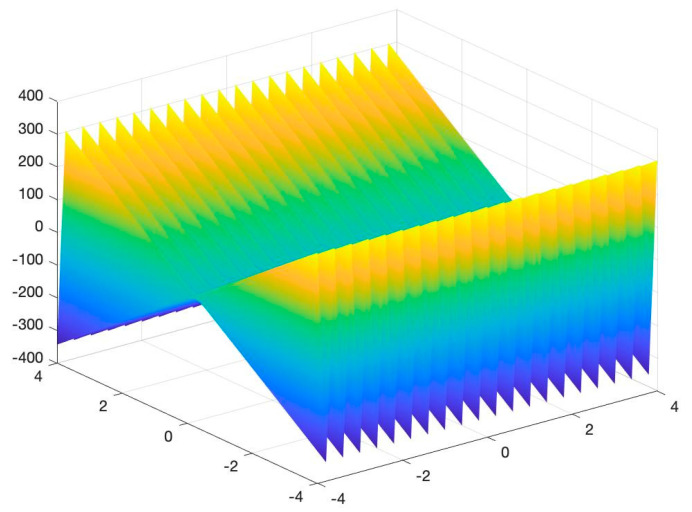
Image of Function 3.

**Figure 5:**
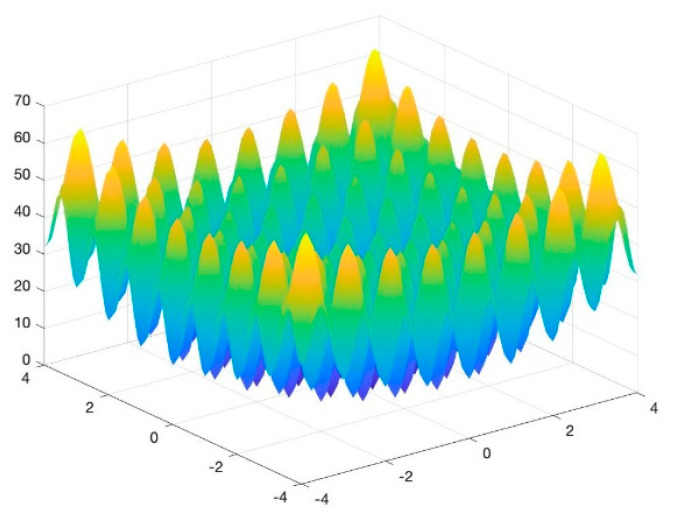
Image of Function 4.

To assess the performance of the proposed algorithm, four benchmarking test functions, as shown in (10)–(13), are utilized, and their images are shown in Figure 2, Figure 3, Figure 4 and Figure 5. The test functions include unimodal functions and multimodal functions with numerous local optimums in their images. Meanwhile, three algorithms—Rao-1, PSO, and the Genetic algorithm (GA)—are benchmarked to assess the performance of the proposed method.

Function 1: Schaffer function

(10) $$minf\left(x,y\right)=0.5-\frac{\left(\sin^{2}\sqrt{x^{2}+y^{2}}-0.5\right)}{{\left[1+0.001\left(x^{2}+y^{2}\right)\right]}^{2}},x_{i}\in \left[-10,10\right]$$

Function 2: Camel function

(11) $$minf\left(x,y\right)=\left(4-2.1x^{2}+\frac{x^{4}}{3}\right)x^{2}+xy+\left(-4+4y^{2}\right)y^{2},\;x,y\in \left[-100,100\right]$$

Function 3:

(12) minf(x,y)=−[xsin(9πy)+ycos(25πx)+20],x,y∈[−10,10]

Function 4:

(13) $$minf\left(x,y\right)=20+x^{2}+y^{2}-10\times \left(\cos2\pi x+\cos2\pi y\right),x_{i}\in \left[-4,4\right]$$

For the above four benchmark functions, four algorithms have experimented 50 times, respectively. According to the results presented in Table 1, The proposed hybrid Rao-NM algorithm achieves the best performance in terms of both efficiency and precision among all considered methods. Besides, though both the Rao-1 algorithm and the proposed hybrid Rao-NM algorithm can quickly converge to the optimal value, the Rao-NM algorithm has higher accuracy, especially for the F2 function, it can precisely converge to the optimal value. For the F3 function, many local optimal values in the solution space exist, and the proposed hybrid algorithm can find the optimal value accurately and efficiently. Therefore, the proposed algorithm outperforms other algorithms in searching for the optimal solution.

### 4.2. Sensitivity Analysis on Controlling Parameters

Since the two controlling parameters, the population size and the number of iterations, are included for all considered TM algorithms, they are tuned based on 368 images selected from the Oxford-IIIT Pet Dataset [39]. Nine parameter configurations are employed, and the grid search is utilized. All considered algorithms are implemented on a PC with AMD Ryzen 9 3950X CPU and 32 GB RAM. The programs are written by Python3, and they are executed o Windows 10. The algorithm-specific parameters of PSO and GA are set as follows:

(1) PSO parameter settings [40]: Cognitive and social acceleration constants *C*1 = 1.8, *C*2 = 1.8, self-weighting factor = 1.0, and independent random numbers *r*1 and *r*2 are distributed in the range of [0, 1].

(2) GA parameter settings [41]: The mutation probability = 0.05, the elite ratio = 0.01, the crossover probability = 0.75, and the parent portion = 0.1.

The matching accuracy and execution time of different algorithms are shown in Table 2, Table 3 and Table 4. In this study, the success rate is defined in (14) to depict the accuracy.

(14) $$\;\;\;R=\frac{T}{S}\times 100\%$$

where *R* is the success rate, T is the number of times that the matching pixel position is the same as the template image in the experiment, and S is the total number of experiments.

### 4.3. Template Matching Results

The Oxford Pets Dataset of 2580 images [39] is utilized to compare the performance of different algorithms based on the optimized parameters. Each algorithm is executed ten times, and the accuracy and execution time are presented in Table 5.

According to Table 5, it can be seen that the proposed method outperforms other benchmarking methods in terms of the highest accuracy and the shorter computing time. Meanwhile, the execution time of the proposed method is slightly longer than that of the Rao-1 algorithm. Thus, it is more practical to apply the proposed method for real applications.

**Figure 6:**
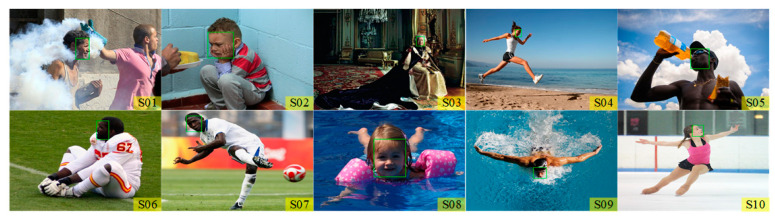
Example images of the WIDER FACE dataset.

To assess the performance of the proposed method on real biometrics recognition tasks, 94 images collected from the V47 dataset [42] and 100 images selected from the WIDER FACE dataset [43] are employed to evaluate the performance of the proposed method on person re-identification and face detection problems. The images from the WIDER FACE dataset are with a high degree of variability in scale, pose, and occlusion, as shown in Figure 6. The image matching results are obtained and shown in Table 6 and Table 7.

According to the results presented in Table 6 and Table 7, the proposed method dominates other methods with the highest accuracy for both two datasets. Therefore, the proposed method is applicable for face detection and person re-identification tasks. Since different scenes are included in these images, TM using the Rao-NM method offers more robust results.

**Figure 7:**
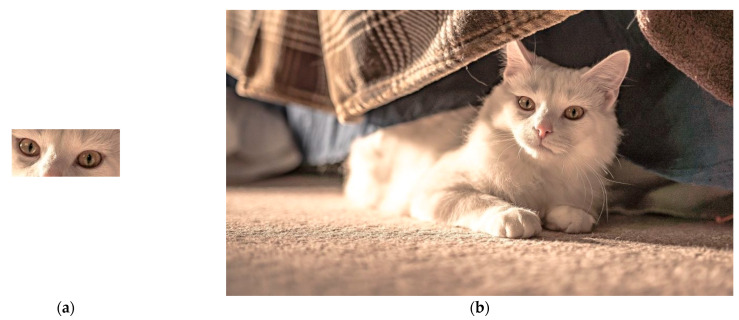

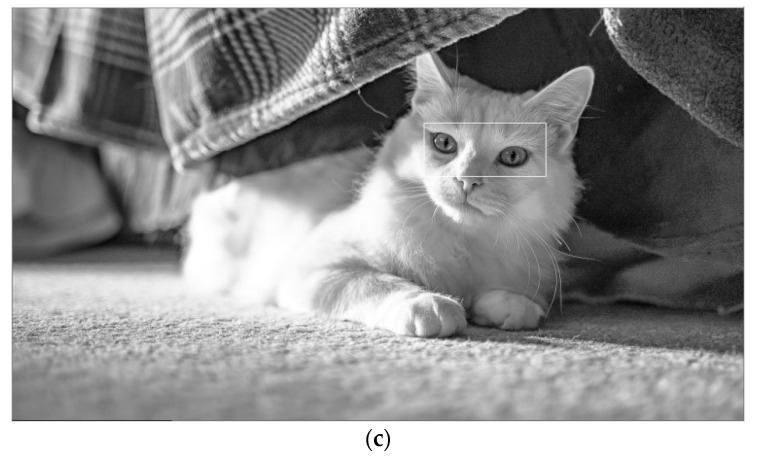
(**a**) Predefined template image (160 x 70); (**b**) source image (960 × 540); (**c**) TM result.

**Figure 8:**
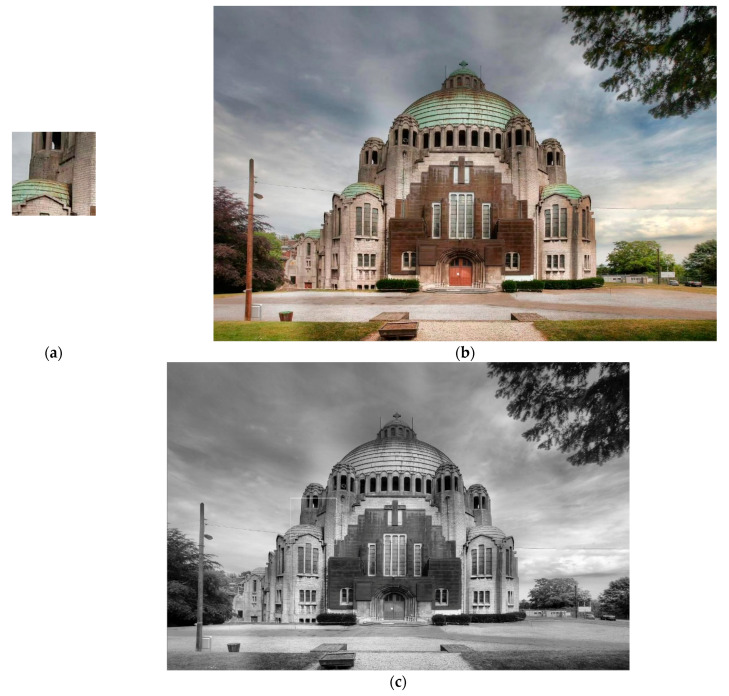
(**a**) Predefined template image (150 × 150); (**b**) source image (1000 × 1500); (**c**) TM result.

**Figure 9:**
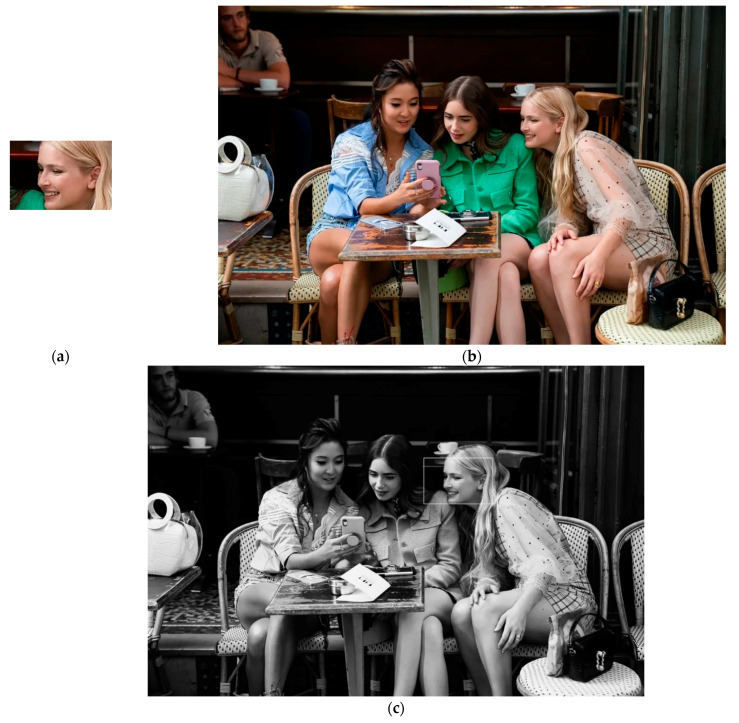
(**a**) Predefined template image (100 × 150); (**b**) source image (1080 × 720); (**c**) TM result.

Three large images of different sizes are employed to validate the actual performance of all considered algorithms, shown in Figure 7, Figure 8 and Figure 9. Each algorithm is executed 50 times independently based on three images to validate their average performance.

Table 8 shows the proposed hybrid Rao-NM algorithm dominates all the compared algorithms in terms of the highest success rate. Although the Rao-1 algorithm requires the least execution time, it performs badly in TM of these three images. Especially, the success rate is only 2% by using the Rao-1 algorithm for Image 2. Thus, it is not suitable to directly apply the Rao-1 algorithm for TM problems. Compared with PSO and GA algorithms, the search efficiency and accuracy of the proposed algorithm are greatly improved over all three images. As shown in Table 8, the hybrid Rao-NM algorithm matching accuracy can reach more than 85%, while PSO and GA algorithms can only offer success rates of less than 85%. The above comparison results show that it is more practical to apply the proposed hybrid algorithm for TM problems.

## 5. Conclusions and Future Work

In this paper, a novel hybrid optimization algorithm, combining the Rao-1 algorithm and the NM method, is proposed to address the image matching problem in an effective and efficient way. The proposed algorithm incorporates the powerful largescale global search ability of the Rao-1 algorithm and the thorough local search capability of the NM method. Thus, the Rao-NM algorithm can accurately search for high-quality optimal solutions.

To verify the robustness and the efficiency of the proposed Rao-NM algorithm, four commonly applied test functions, and three image datasets are utilized. Meanwhile, three benchmarking algorithms are considered. The experimental results demonstrate that the proposed algorithm is more accurate than other recently reported algorithms and takes less time to converge to the optimum. Considering the higher accuracy and shorter execution time, the proposed algorithm is practical for image matching problems.

The proposed method is implemented serially on the CPU. Since current image processing and computer vision algorithms can run on modern GPUs, the parallel version of the proposed method will be investigated, and thus, the multi-core CPUs and many-core GPUs can be employed to speed up the image matching task. Meanwhile, the elite mechanism can be incorporated into the Rao-1 algorithm to improve the global searchability.

## Figures and Tables

**Table 1:** Results comparisons of the benchmark.

Algorithm		F1	F2	F3	F4
	Theoretical Optimal Value	0.0	−1.0316	−39.9450	0.0
Rao-1	Average time	6.9750 × 10^−6^	8.8250 × 10^−6^	0.0032	0.0033
	Actual optimal	0.0610	−0.1943	−39.8498	0.0003
PSO	Average time	0.2421	0.2767	0.3727	0.2229
	Actual optimal	0.0048	57.6269	−39.0897	2.6623
GA	Average time	1.2741	1.2757	1.2739	1.2990
	Actual optimal	0.0024	−0.9549	−39.4269	0.0032
Rao-NM	Average time	4.1650 × 10^−5^	3.7300 × 10^−5^	0.0032	0.0033
	Actual optimal	**0.0025**	**−1.0316**	**−39.8500**	**5.2560 × 10^−6^**

The bold indicates the best results.

**Table 2:** Test results of TM using Rao-NM algorithm.

Population Size	No. of Iterations	R	Time (s)
50	50	77.71%	71.42
50	100	80.70%	140.23
50	200	84.51%	277.56
100	50	85.32%	138.61
100	100	89.13%	274.53
100	200	87.77%	546.94
200	50	88.58%	273.82
200	100	91.84%	544.45
200	200	95.10%	1085.16

**Population Size**

**No. of Iterations**

**R**

**Time (s)**

**Table 3:** Test results of TM using PSO.

Population Size	No. of Iterations	R	Time (s)
50	50	24.45%	99.38
50	100	29.89%	196.78
50	200	41.57%	371.76
100	50	32.06%	197.40
100	100	48.36%	311.62
100	200	61.68%	622.31
200	50	52.44%	372.62
200	100	66.03%	624.34
200	200	77.44%	1194.61

**Table 4:** Test results of TM using GA.

Population Size	No. of Iterations	R	Time (s)
50	50	15.48%	196.89
50	100	34.51%	394.18
50	200	58.96%	776.82
100	50	35.05%	399.27
100	100	66.03%	792.42
100	200	82.06%	1567.83
200	50	67.39%	798.36
200	100	88.31%	1570.10
200	200	94.29%	2951.44

**Table 5:** Performance of different methods on the Oxford Pets Dataset.

Model	R (%)	Time (s)
PSO	49.76 ± 0.84	2616.38 ± 9.29
GA	70.17 ± 0.82	4345.63 ± 151.69
Rao-1	54.17 ± 0.59	1666.08 ± 25.15
Proposed	88.94 ± 0.64	1807.25 ± 30.69

**Table 6:** Performance of different methods for Person Re-identification.

Model	R (%)	Time (s)
PSO	16.91 ± 3.34	97.067 ± 0.61
GA	48.19 ± 3.54	151.736 ± 4.06
Rao-1	19.68 ± 1.60	86.23 ± 0.55
Proposed	56.70 ± 3.13	89.11 ± 1.09

**Table 7:** Performance of different methods for FaceDetector.

Model	R (%)	Time (s)
PSO	15.2 ± 2.03	126.533 ± 1.51
GA	44.3 ± 4.59	189.719 ± 2.26
Rao-1	19.5 ± 1.50	116.022 ± 0.54
Proposed	67.1 ± 3.95	120.916 ± 0.67

**Table 8:** Results comparisons of TM.

		Image 1	Image 2	Image 3
PSO	Average time	5.64	24.44	27.86
	Accuracy	34%	28%	50%
GA	Average time	9.33	14.75	24.59
	Accuracy	82%	84%	50%
Rao-1	Average time	4.27	13.86	11.72
	Accuracy	34%	2%	52%
Rao-NM	Average time	4.28	13.87	11.73
	Accuracy	96%	86%	86%
